# Isolation, characterization, and genetic manipulation of cold-tolerant, manganese-oxidizing *Pseudomonas* sp. strains

**DOI:** 10.1128/aem.00510-24

**Published:** 2024-08-30

**Authors:** Ian Jones, Duncan Vermillion, Chase Tracy, Robert Denton, Rick Davis, Kati Geszvain

**Affiliations:** 1 Department of Biological Sciences, California State University, Chico, California, USA; 2 Division of Science and Math, University of Minnesota, Morris, Minnesota, USA; 3 Department of Biology, Marian University, Indianapolis, Indiana, USA; 4 Texas State University, NASA Johnson Space Center, Houston, Texas, USA; Colorado School of Mines, Golden, Colorado, USA

**Keywords:** manganese oxidation, *Pseudomonas*, psychrotolerant, compost

## Abstract

Manganese-oxidizing bacteria (MnOB) produce Mn oxide minerals that can be used by humans for bioremediation, but the purpose for the bacterium is less clear. This study describes the isolation and characterization of cold-tolerant MnOB strains isolated from a compost pile in Morris, Minnesota, USA: *Pseudomonas* sp. MS-1 and DSV-1. The strains were preliminarily identified as members of species *Pseudomonas psychrophila* by 16S rRNA analysis and a multi-locus phylogenetic study using a database of 88 genomes from the *Pseudomonas* genus. However, the average nucleotide identity between these strains and the *P. psychrophila* sp. CF149 type strain was less than 93%. Thus, the two strains are members of a novel species that diverged from *P. psychrophila*. DSV-1 and MS-1 are cold tolerant; both grow at 4°C but faster at 24°C. Unlike the mesophilic MnOB *P. putida* GB-1, both strains are capable of robustly oxidizing Mn at low temperatures. Both DSV-1 and MS-1 genomes contain homologs of several Mn oxidation genes found in *P. putida* GB-1 (*mnxG*, *mcoA*, *mnxS1*, *mnxS2,* and *mnxR*). Random mutagenesis by transposon insertion was successfully performed in both strains and identified genes involved in Mn oxidation that were similar to those found in *P. putida* GB-1. Our results show that MnOB can be isolated from compost, supporting a role for Mn oxidation in plant waste degradation. The novel isolates *Pseudomonas* spp. DSV-1 and MS-1 both can oxidize Mn at low temperature and likely employ similar mechanisms and regulation as *P. putida* GB-1.

**IMPORTANCE:**

Biogenic Mn oxides have high sorptive capacity and are strong oxidants. These two characteristics make these oxides and the microbes that make them attractive tools for the bioremediation of wastewater and contaminated environments. Identifying MnOB that can be used for bioremediation is an active area of research. As cold-tolerant MnOB, *Pseudomonas* sp. DSV-1 and MS-1 have the potential to expand the environmental conditions in which biogenic Mn oxide bioremediation can be performed. The similarity of these organisms to the well-characterized MnOB *P. putida* GB-1 and the ability to manipulate their genomes raise the possibility of modifying them to improve their bioremediation ability.

## INTRODUCTION

The transition metal manganese (Mn) is an essential micronutrient found in the Earth’s crust. As a transition metal, it can be found present in the environment in several possible redox states, including the soluble, reduced form Mn(II) and insoluble Mn(IV), which form Mn oxide minerals. While oxidation of Mn(II) to Mn(IV) can occur abiotically, microorganisms can increase the rate of reaction substantially (1). These organisms include the white rot fungi (2–4) and a wide variety of bacteria, including both gram-positive and gram-negative representatives (5, 6). The metabolic function of Mn oxidation is unclear. The resulting minerals may protect cells from environmental hazards, such as oxidative stress (7). Alternatively, the reactive Mn oxide minerals may allow the bacteria to degrade complex organics to digestible byproducts; the minerals themselves can serve as reservoirs of organic carbon (8, 9). Work from Yu and Leadbetter has shown that some species of bacteria can derive energy directly from the thermodynamically favorable oxidation of Mn (10).

In the environment, Mn oxidation may play a role in the breakdown of plant material. The concentration and redox state of Mn in leaf litter strongly correlate with the rate of litter decomposition (11), and the ability of forest ecosystems to store carbon is negatively correlated with Mn concentration (12). A significant component of plant litter is the cell wall component lignin. Lignin is a large, three-dimensional polymer of phenylpropanoid subunits; its large size and irregular structure render it, especially, difficult to degrade enzymatically (13). However, several species of fungus and bacteria are capable of lignin degradation (13–15). These organisms employ both laccase enzymes and a variety of heme-containing peroxidase enzymes, including Mn peroxidase. A major mechanism by which lignin-degrading enzymes work is through the production of soluble Mn(III) species via an oxidation reaction (15, 16).

Biogenic Mn oxides (BMO) and Mn-oxidizing bacteria (MnOB) are actively being investigated for their possible applications in bioremediation due to the highly reactive and sorptive nature of the BMO. BMO generated by *E. coli* cells genetically modified to express a non-blue laccase from *Bacillus* sp. GZB have been shown to degrade the endocrine disruptor bisphenol A (17). BMO from the naturally Mn-oxidizing strain *Pseudomonas* sp. QJX-1 can degrade the herbicide glyphosate, and the bacteria can use the resulting breakdown products as a carbon, phosphate, or nitrogen source (18). Oxidation of pollutants is not the only mechanism of bioremediation by BMO. They have also been shown to remove arsenic from wastewater through precipitation of metal arsenates or adsorption on ferromanganese minerals (19). Breakdown of 17α-ethinylestradiol (EE2) by BMO was increased 15-fold by the presence of the MnOB *Pseudomonas putida* MnB1 (20). Thus, optimal bioremediation may require living MnOB, not just the oxides they produce, making it important to identify MnOB that can thrive under a variety of growth conditions.

One of the best studied MnOB is *Pseudomonas putida* GB-1. This gram-negative gamma-proteobacterium has been shown to possess three genes encoding Mn oxidase enzymes that each appear to oxidize Mn(II) to Mn(IV). Two of the oxidases belong to the multi-copper oxidase family of enzymes, encoded by the genes *mnxG* and *mcoA* (21). The third oxidase, MopA, is an animal heme peroxidase (22). Mn oxidation in this species is also dependent on a two-component regulatory pathway comprising two sensor kinases —MnxS1 and MnxS2—and a σ^54^-dependent response regulator MnxR (23). Regulation of Mn oxidation in this species appears to be linked to the motile vs biofilm lifestyle switch since the deletion of regulatory gene *fleQ* results in altered Mn oxidation (22, 24).

If a physiological function of Mn oxidation is the breakdown of recalcitrant organic carbon (ROC) for use as a food source, MnOB would be predicted to be found in areas with high concentrations of ROC, such as a compost pile. Sampling a compost pile on the campus of the University of Minnesota, Morris successfully resulted in the isolation of two MnOB strains, *Pseudomonas* sp. DSV-1 and MS-1. Both strains exhibit cold-tolerant growth and manganese oxidation down to 4°C. Genome sequence shows the two strains are very similar and have genes identified as important for Mn oxidation in the well-characterized MnOB *Pseudomonas putida* GB-1. We further demonstrate that the two strains can be genetically manipulated, illustrating the possibility of using these strains for bioremediation and studies of the evolution of Mn oxidation in the pseudomonads.

## RESULTS

### Isolation and identification of DSV-1 and MS-1

**Fig 1:**
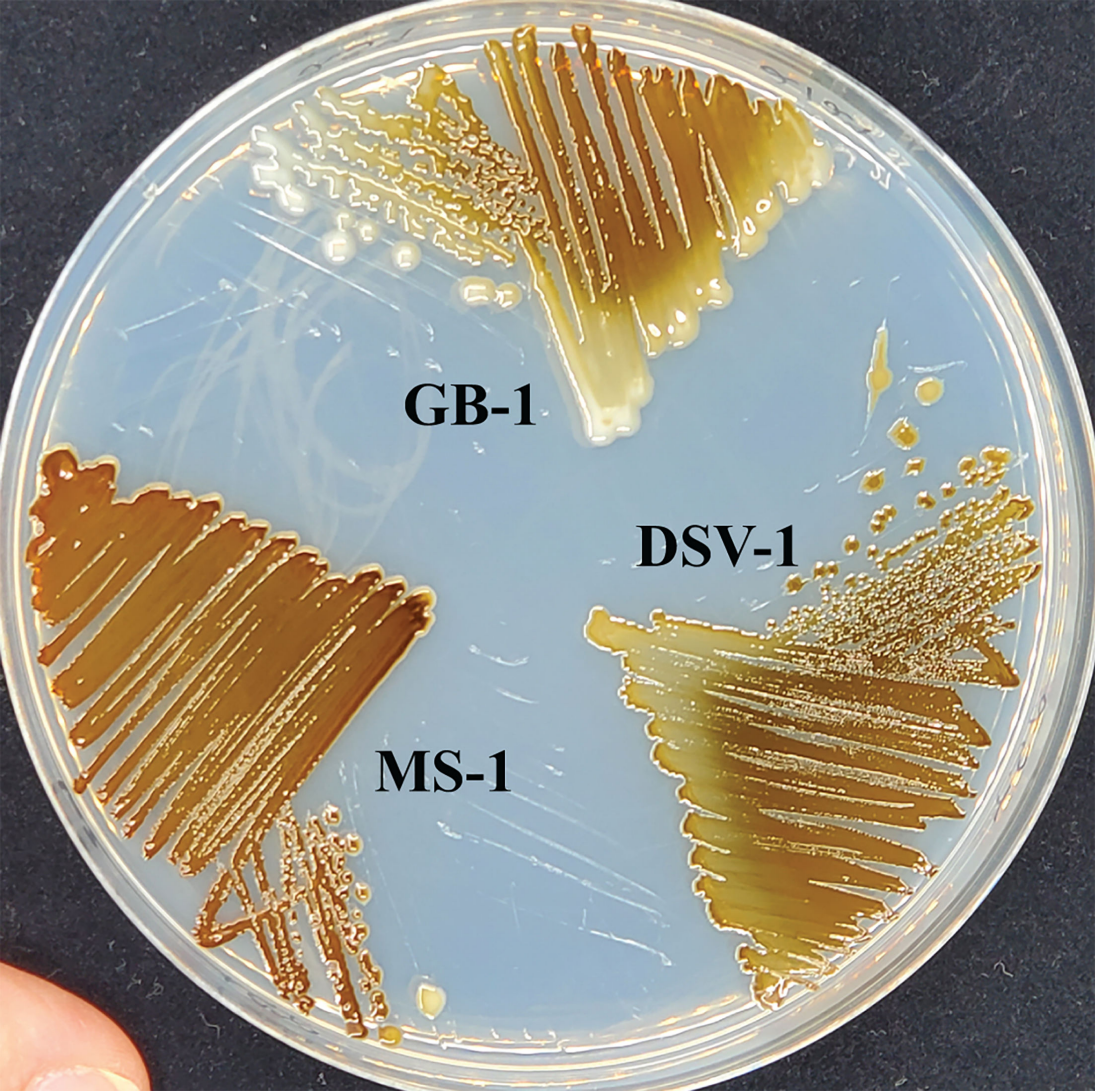
Mn oxidation phenotype of new isolates compared to *Pseudomonas putida* GB-1. Strains were streaked onto Lept media and incubated at 24°C for 3 days. GB-1, *Pseudomonas putida* GB-1; MS-1, *Pseudomonas* sp. MS-1; DSV-1, *Pseudomonas* sp. DSV-1.

To test the prediction that Mn oxidation allows bacteria to degrade plant matter, samples were taken from a compost pile consisting of an approximate ratio of 75% plant material and 25% food waste (Troy Otsby, personal communication). Three samples were taken from the surface of the pile and at depths of ~15 cm and ~30 cm. From an initial set of ~10 Mn-oxidizing candidate species, four isolates were purified, and isolates MS-1 and DSV-1 were chosen for further characterization. At 24°C on solid media, both develop the brown colony color indicative of Mn oxidation (Fig. 1). The presence of Mn oxides was confirmed using leucoberbelin blue [LBB; data not shown, (25, 26)]. This oxidation behavior is similar to the well-characterized MnOB *P. putida* GB-1 (GB-1, Fig. 1). To tentatively identify the isolates, their 16S rRNA gene was amplified by colony PCR and sequenced. The resulting sequences were 100% identical to each other, and the best match in GenBank was to *Pseudomonas psychrophila* type strain E-3 (27) (NR_028619.1, 99% coverage, 99.66% identity).

### Phylogenetic tree of *Pseudomonas* genus

**Fig 2:**
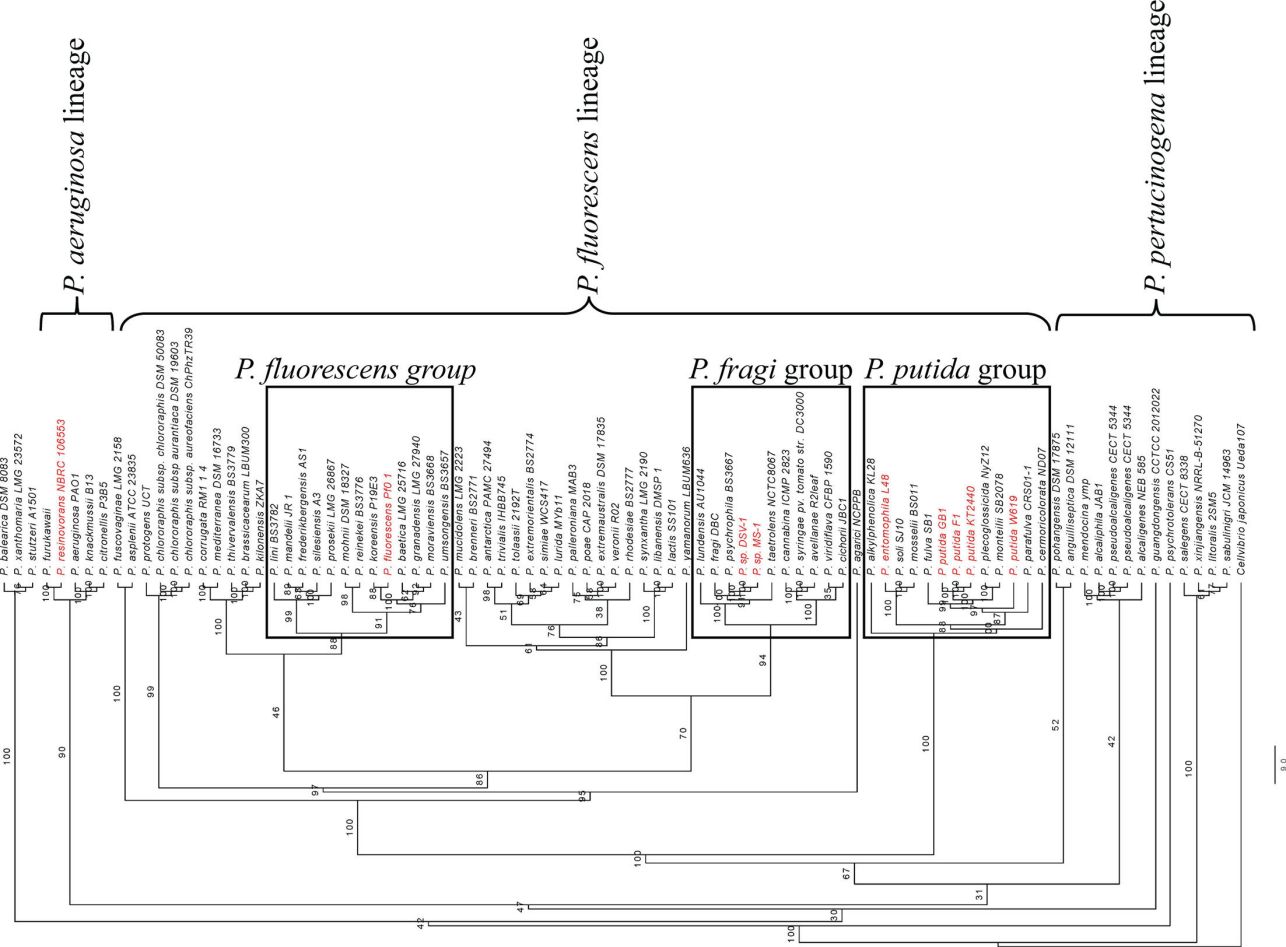
Phylogenetic tree of genus *Pseudomonas*. A total of 88 species of the genus *Pseudomonas* are represented by this tree, which uses a concatenated sequence of the 16S rRNA gene, *rpoB*, *rpoD*, and *gyrB* to construct proposed evolutionary relationships. Known MnOB are highlighted in red.

To further investigate the relationship of DSV-1 and MS-1 to other pseudomonads, multi-locus sequence alignment was performed to construct a phylogenetic tree comparing 88 species of the *Pseudomonas* genus and the new isolates (Table 1). The species chosen represent members of each of the major clades in the genus: the *fluorescens*, *aeruginosa*, and *pertucinogena* lineages (28). MS-1 and DSV-1 group with *P. psychrophila* and *P. fragi* in the *P. fragi* group within the *fluorescens* lineage (Fig. 2). This lineage not only contains other known MnOB, *P. putida* GB-1, *P. entomophila* L48, and *P. fluorescens* PfO-1, but also species not known to oxidize Mn, including *P. syringae* pv. tomato str DC3000 (21).

**TABLE 1:** Accession numbers of strains used in phylogenetic tree

Species name	Accession number	Sequence type
*Cellvibrio japonicus* Ueda107	NC_010995	Complete genome
*Pseudomonas* sp. DSV-1	JAYMYG000000000	Complete genome
*Pseudomonas* sp. MS-1	JAYMYG000000000	Complete genome
*Pseudomonas aeruginosa* PAO1	NC_002516.2	Complete genome
*Pseudomonas agarici* NCPPB 2472	NC_002516.2	Complete genome
*Pseudomonas alcaligenes* NEB 585	NZ_CP014784	Complete genome
*Pseudomonas alcaliphila* JAB1	NZ_CP016162	Complete genome
*Pseudomonas alkylphenolica* KL28	NZ_CP009048	Complete genome
*Pseudomonas anguilliseptica* DSM 12111	NZ_FNSCO00000000	Complete genome
*Pseudomonas antarctica* PAMC 27494	NZ_CP015600	Complete genome
*Pseudomonas asplenii* ATCC 23835	NZ_LT629777	Genome assembly
*Pseudomonas avellanae* R2leaf	NZ_CP026562	Complete genome
*Pseudomonas baetica* LMG 25716	NZ_PHHE00000000	Shotgun sequence
*Pseudomonas balearica* DSM 6083	NZ_CP007511	Complete genome
*Pseudomonas brassicacearum* LBUM 300	NZ_CP012680	Complete genome
*Pseudomonas brenneri*	NZ_LT629800	Genome assembly
*Pseudomonas cannabina* ICMP 2823	NZ_FNKU00000000	Shotgun sequence
*Pseudomonas cedrina* BS2981	NZ_LT629753	Genome assembly
*Pseudomonas cerasi* PL963	NZ_LT963395	Genome assembly
*Pseudomonas chlororaphis* subsp*. aurantiaca* DSM 19603	NZ_CP027746	Complete genome
*Pseudomonas chlororaphis* subsp*. aureofaciens* ChPhzTR39	NZ_CP027749	Complete genome
*Pseudomonas chlororaphis* subsp*. chlororaphis* DSM 50083	NZ_CP027712	Complete genome
*Pseudomonas cichorii* JBC1	NZ_CP007039	Complete genome
*Pseudomonas citronellolis* P3B5	NZ_CP014158	Complete genome
*Pseudomonas corrugata* RM1-1-4	NZ_CP014262	Complete genome
*Pseudomonas cremoricolorata* ND07	NZ_CP009455	Complete genome
*Pseudomonas entomophila* L48	NC_008027	Complete genome
*Pseudomonas extremaustralis* DSM 17835	NZ_LT629689	Genome assembly
*Pseudomonas extremorientalis* BS2774	LT629708	Genome assembly
*Pseudomonas fluorescens* Pf0-1	NC_007492	Complete genome
*Pseudomonas fragi* DBC	NZ_CP021986	Complete genome
*Pseudomonas frederiksbergensis* AS1	NZ_CP018319	Complete genome
*Pseudomonas fulva* SB1	NZ_CP023048	Complete genome
*Pseudomonas furukawaii*	NZ_AP014862	Complete genome
*Pseudomonas fuscovaginae* LMG 2158	NZ_LT629972	Genome assembly
*Pseudomonas granadensis* LMG 27940	NZ_LT629778	Genome assembly
*Pseudomonas guangdongensis* CCTCC 2012022	NZ_LT629780	Genome assembly
*Pseudomonas kilonensis* ZKA7	NZ_QEKL00000000	Shotgun sequence
*Pseudomonas knackmussii* B13	NZ_HG322950	Complete genome
*Pseudomonas koreensis* P19E3	NZ_CP027477	Complete genome
*Pseudomonas lactis* SS101	NZ_CM001513	Shotgun sequence
*Pseudomonas libanensis* DMSP-1	NZ_CP034425	Complete genome
*Pseudomonas lini* BS3782	NZ_LT629746	Genome assembly
*Pseudomonas litoralis* 2SM5	NZ_LT629748	Genome assembly
*Pseudomonas lundensis* AU1044	CP017687	Complete genome
*Pseudomonas lurida* MYb11	NZ_CP023272	Complete genome
*Pseudomonas mandelii* JR-1	NZ_CP005960	Complete genome
*Pseudomonas mediterranea* DSM 16733	NZ_LT629790	Genome assembly
*Pseudomonas mendocina* ymp	NC_009439	Complete genome
*Pseudomonas mohnii* DSM 18327	NZ_FNRV00000000	Shotgun sequence
*Pseudomonas monteilii* SB3078	NC_023075	Complete genome
*Pseudomonas moraviensis* BS3668	NZ_LT629788	Genome assembly
*Pseudomonas mosselii* BS011	NZ_CP023299	Complete genome
*Pseudomonas mucidolens* LMG 2223	NZ_LT629802	Genome assembly
*Pseudomonas palleroniana* MAB3	NZ_CP025494	Complete genome
*Pseudomonas parafulva* CRS01-1	NZ_CP009747	Complete genome
*Pseudomonas plecoglossicida* NyZ12	NZ_CP010359	Complete genome
*Pseudomonas poae* CAP-2018	CP034537	Complete genome
*Pseudomonas pohangensis* DSM 17875	NZ_LT629785	Genome assembly
*Pseudomonas prosekii* LMG 26867	NZ_LT629762	Genome assembly
*Pseudomonas protegens* UCT	CP017964	Complete genome
*Pseudomonas pseudoalcaligenes* CECT 5344	NZ_HG916826	Complete genome
*Pseudomonas psychrophila* BS3667	LT629795	Genome assembly
*Pseudomonas psychrotolerans* CS51	NZ_CP021645	Complete genome
*Pseudomonas putida* F1	CP000712	Complete genome
*Pseudomonas putida* GB-1	NC_010322	Complete genome
*Pseudomonas putida* KT2440	NC_002947	Complete genome
*Pseudomonas putida* W619	NC_010501	Complete genome
*Pseudomonas reinekei* BS3776	NZ_LT629709	Genome assembly
*Pseudomonas resinovorans* NBRC 106553	NC_021499	Complete genome
*Pseudomonas rhodesiae* BS2777	NZ_LT629801	Genome assembly
*Pseudomonas sabulinigri* JCM 14963	NZ_LT629763	Genome assembly
*Pseudomonas salegens* CECT 8338	NZ_LT629787	Genome assembly
*Pseudomonas silesiensis* A3	NZ_CP014870	Complete genome
*Pseudomonas simiae* WCS417	NZ_CP007637	Complete genome
*Pseudomonas soli* SJ10	NZ_CP009365	Complete genome
*Pseudomonas stutzeri* A1501	NC_009434	Complete genome
*Pseudomonas synxantha* LMG 2190	NZ_LT629786	Genome assembly
*Pseudomonas syringae* pv. tomato str. DC3000	AE016853	Complete genome
*Pseudomonas taetrolens* NCTC8067	NZ_LR134393	Genome assembly
*Pseudomonas thivervalensis* BS3779	NZ_LT629691	Genome assembly
*Pseudomonas tolaasii* 2192T	NZ_CP020369	Complete genome
*Pseudomonas trivialis* IHBB745	NZ_CP011507	Complete genome
*Pseudomonas umsongensis* BS3657	NZ_LT629767	Genome assembly
*Pseudomonas veronii* R02	NZ_CP018420	Complete genome
*Pseudomonas viridiflava* CFBP 1590	NZ_LT855380	Genome assembly
*Pseudomonas xanthomarina* LMG 23572	NZ_LT629970	Genome assembly
*Pseudomonas xinjiangensis* NRRL B-51270	NZ_LT629736	Genome assembly
*Pseudomonas yamanorum* LBUM636	NZ_CP012400	Complete genome

### Complete genome sequence of MS-1 and DSV-1

Using a combination of Illumina and Nanopore sequencing approaches, complete genome sequences were generated for MS-1 (NCBI accession #: JAYMYF000000000) and DSV-1 (NCBI accession #: JAYMYG000000000). The MS-1 genome is smaller than DSV-1 (5.3 vs 5.7 Mb), with fewer predicted genes (Table 2). Genetic relatedness and species attribution can be determined using the genome-wide average nucleotide identity (gANI) and alignment fraction (AF) metrics (29–31). Using tools available at the Integrated Microbial Genomes and Microbiomes website at the Joint Genome Institute (https://img.jgi.doe.gov/) (32), it was determined that the DSV-1 and MS-1 pairwise gANI is 99.1% and 88.6% for AF (Table 3), supporting their identification as two strains within the same species. However, their best match to a *P. psychrophila* strain was *P. psychrophila* CF149 with an gANI of 92.6%–92.8% and an AF of 85.8%–87.4% (Table 5). Because a gANI of >95% is commonly used to assign strains to the same species, DSV-1 and MS-1 have diverged sufficiently from *P. psychrophila* to be considered members of a different species.

**TABLE 2:** Comparison of GB-1, MS-1, and DSV-1 genomes

Organism	GB-1	DSV-1	MS-1
No. of scaffolds	1	5	7
Bases	6,078,430	5,720,809	5,342,990
% GC	61.94	57.61	57.90
CDS	5,515	5,391	5,144
Percent w/ funct. prediction	76.1	77.6	80.5
Cold shock genes (COG1278, *cspA*)	6	6	6

**TABLE 3:** Average nucleotide identity and alignment fraction

Genome 1 name, ID #	Genome 2 name, ID #	ANI1- >2	ANI2- >1	AF1- >2	AF2- >1
*Pseudomonas* DSV-1, 2986949986	*Pseudomonas* MS-1, 2986955527	99.1	99.1	88.6	88.6
*Pseudomonas* MS-1, 2986955527	*P. psychrophila* CF149, 2541047098	92.8	92.8	85.8	85.8
*Pseudomonas* DSV-1, 2986949986	*P. psychrophila* CF149, 2541047098	92.6	92.6	87.4	87.4

### Identification of putative Mn oxidation genes

Using the genome sequences, it was possible to identify orthologs of Mn oxidation genes from the well-characterized MnOB *P. putida* GB-1. This organism encodes three separate Mn oxidase enzymes in its genome, *mnxG*, *mcoA,* and *mopA* (21, 22). MS-1 and DSV-1 both carry homologs to *mnxG* and *mcoA* but not *mopA* (Table 4). Three genes predicted to encode parts of a two-component regulatory pathway*—mnxS1*, *mnxS2,* and *mnxR*—are also essential for Mn oxidation in *P. putida* GB-1 (23). Each of these genes are present in both DSV-1 and MS-1 (Table 4).

**TABLE 4:** Putative Mn oxidation gene orthologs in DSV-1 and MS-1

*P. putida* GB-1 Mn oxidation gene	Predicted function	DSV-1 IMG gene ID # (% coverage/% identity)	MS-1 IMG gene ID# (% coverage/% identity)
*mnxG*, 641547570	Mn oxidase enzyme (21)	2986952721 99%/79.2%	2986957664 99%/79.3%
641547571	40-Residue YVTN family beta-propeller repeat protein	2986952720 98%/59.7%	2986957665 98%/59.7%
641547572	Electron transport protein SCO1/SenC	2986952719 99%/60.4%	2986957666 99%/60.4%
641547573	Electron transport protein SCO1/SenC	2986952718 95%/64.15%	2986957667 95%/64.15%
641547574	Amino acid/amide ABC transporter substrate-binding protein, cytochrome c domain	2986952717 90%/50.6%	2986957668 90%/50.6%
641547575	SurA domain, porin chaperone	2986952716 98%/51.55%	2986957669 98%/51.55%
*mcoA*, 641547786	Mn oxidase enzyme (21)	2986952697 80%/74.1%	2986957688 80%/74.0%
641547787	Electron transport protein SCO1/SenC	2986952696 96%/59.6%	2986957689 96%/59.4%
641547788	Diguanylate cyclase/phosphodiesterase	2986950947 55%/40.1%	2986959393 55%/40.1%
641547789	ABC transporter, phosphonate, periplasmic substrate-binding protein	2986955028 90%/26.0%	2986958910 12%/39%
641547790	Phage integrase family	2986955374 96%/65.9%	2986958017 93%/63.6%
*mopA*, 641548469	Mn oxidase enzyme (22)	2986953309 2%/21.0%	2986957671 3%/23.5%
*mnxS1*, 641547642	Sensor kinase required for Mn oxidation (23)	2986952698 97%/61.2%	2986957687 97%/61.2%
*mnxS2*, 641547643	Sensor kinase required for Mn oxidation (23)	2986952695 98%/61.8%	2986957690 98%/61.8%
*mnxR*, 641547644	Response regulator required for Mn oxidation (23)	2986952694 100%/77.3%	2986957691 100%/77.3%

**Fig 3:**
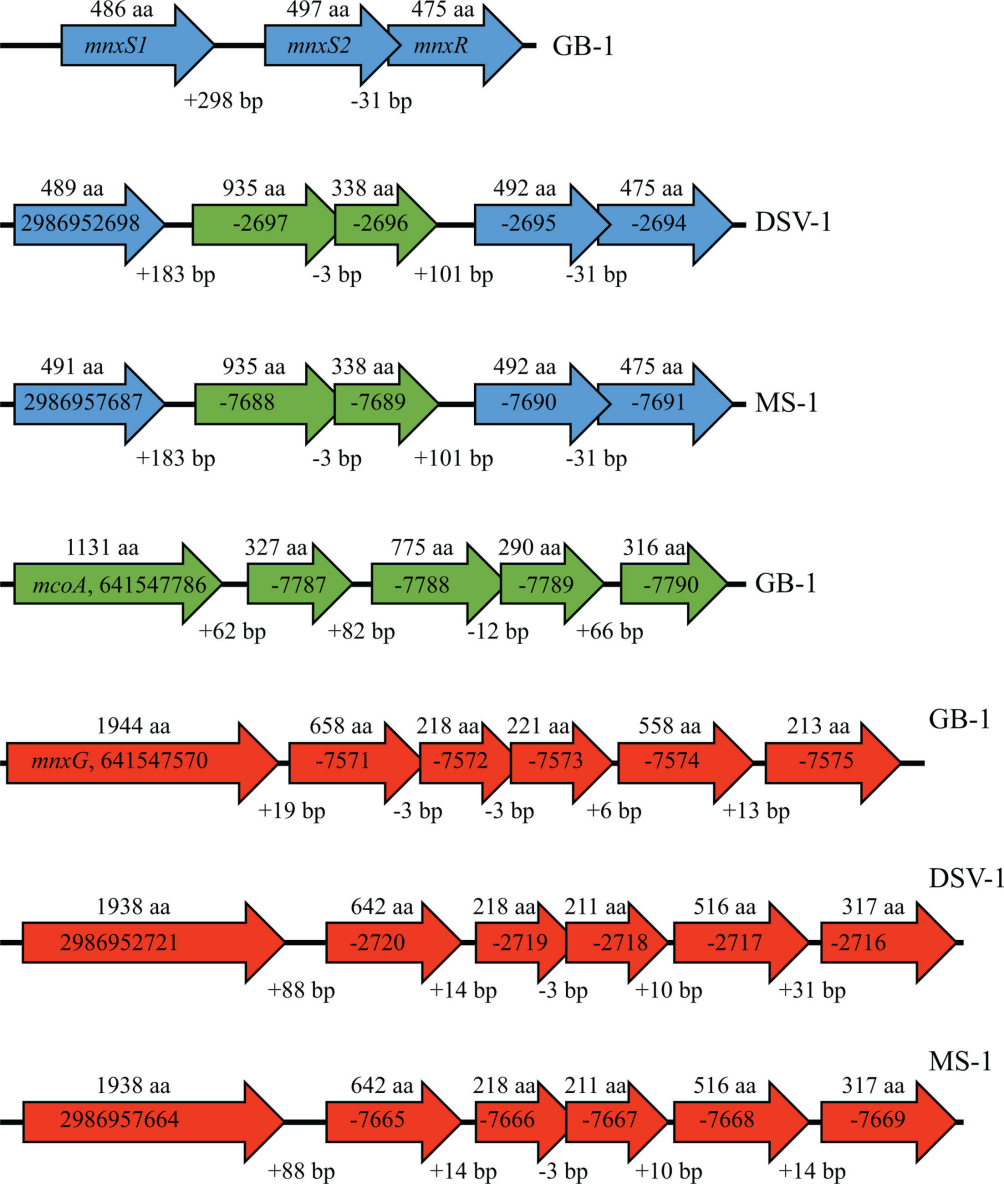
Conservation of putative Mn oxidation operons. Arrows represent predicted genes. Numbers below the arrows represent the number of base pairs (bp) between predicted genes; numbers above the arrows are the length of the predicted protein product in amino acids (aa). Written within the arrows are the gene names and/or IMG gene ID# for the gene. Genes in the putative *mnxG* operon are red, genes in the *mcoA* operon are green, and those in the *mnx* two-component regulatory pathway are blue. GB-1, *Pseudomonas putida* GB-1; MS-1, *Pseudomonas* sp. MS-1; DSV-1, *Pseudomonas* sp. DSV-1.

The spacing between the genes and their orientation on the *P. putida* GB-1 chromosome suggests that *mnxG* and *mcoA* represent the first gene of two operons, respectively, while *mnxS1*, *mnxS2,* and *mnxR* form a third putative operon (23) (Fig. 3). All six of the putative *mnxG* operon genes are found in both DSV-1 and MS-1, and they are found in the same orientation and organization on the chromosome (Table 4; Fig. 3). The *mcoA* putative operon contains five genes in *P. putida* GB-1 (Fig. 3); however, only *mcoA* and the gene immediately downstream, a predicted SCO1/SenC copper chaperone, are conserved in DSV-1 and MS-1 (Table 4; Fig. 3). The genome organization of *mnxS1/S2/R* is also somewhat conserved between GB-1, MS-1, and DSV-1, except that the *mcoA* SCO1/SenC gene pair of the putative *mcoA* operon is located in the space between *mnxS1* and *mnxS2* in both MS-1 and DSV-1 (Fig. 3).

### *Pseudomonas* sp. MS-1 and DSV-1 growth at low temperature

**Fig 4:**
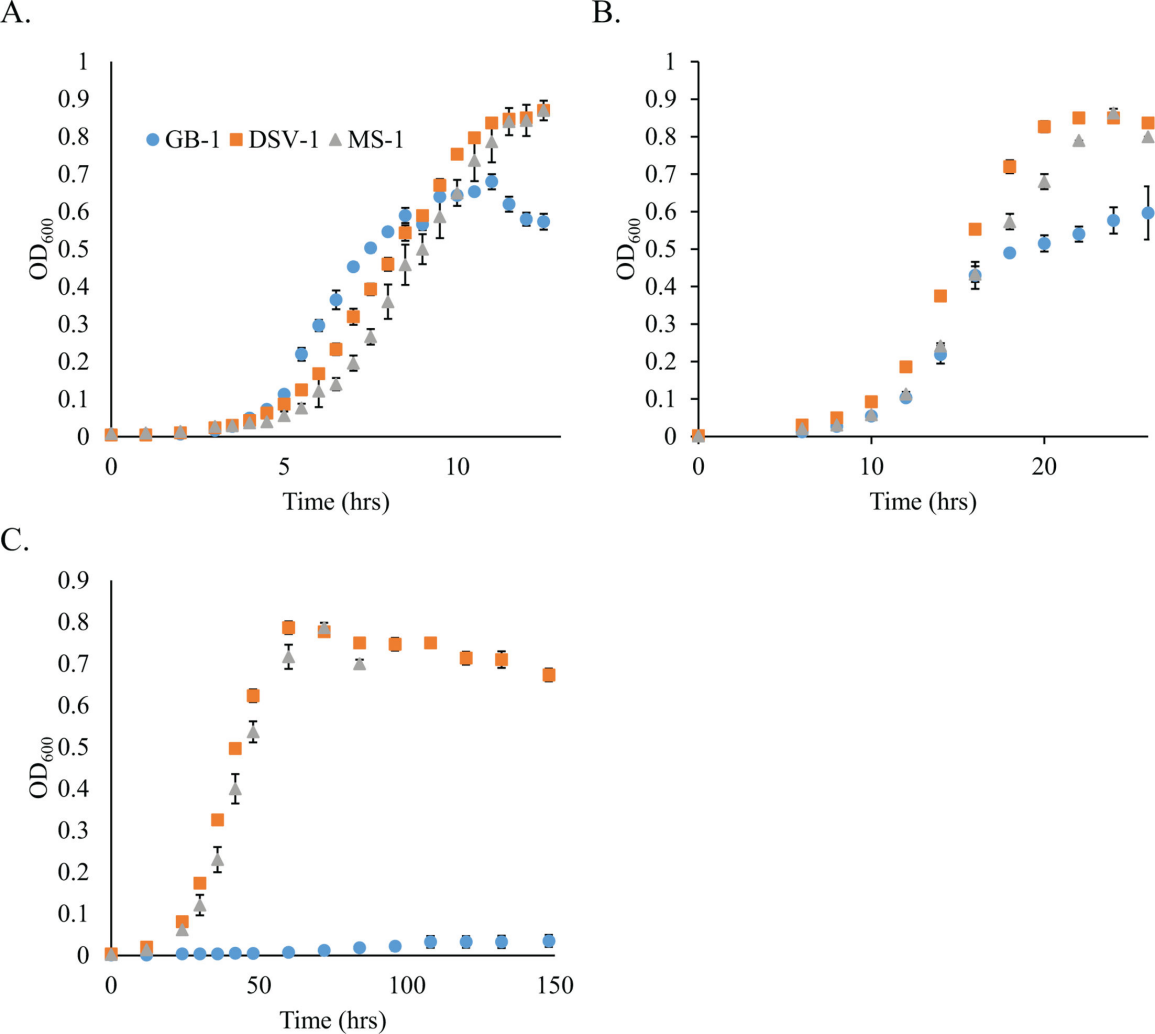
Growth of *P. putida* GB-1, and *P. psychrophila* strains DSV-1 and MS-1 at various temperatures. Datapoints represent the average of three replicates; error bars are the standard deviation. (A) 24°C, (B) 14°C, and (C) 4°C. After 80 h of growth at 4°C, cellular aggregation in the MS-1 culture made it difficult to measure OD_600_.

Because of the close association of DSV-1 and MS-1 to *P. psychrophila* (Fig. 2), a psychrophilic species that can grow at temperatures as low as −1°C (27), we compared the ability of DSV-1 and MS-1 to grow at low temperature to that of the model MnOB *P. putida* GB-1. At 24°C, GB-1 grew somewhat faster than either strain (Table 5), although it reached a lower final optical density (Fig. 4). Growth slowed for all three strains at 14°C (Fig. 4), with GB-1 still doubling at a slightly faster rate than MS-1 or DSV-1 (Table 5). However, at 4°C, GB-1 grew very slowly, with roughly a 24-h doubling time (Fig. 4). Both MS-1 and DSV-1 grew detectably at this low temperature, with doubling times of 7.5 and 8.1 h, respectively (Table 5). Therefore, MS-1 and DSV-1 are capable of growth at low temperature but grow more slowly than at more moderate temperatures. During growth at 4°C, the optical density of the MS-1 culture dropped dramatically once the culture reached stationary phase. This could suggest a defect in survival at low temperature for this strain. However, the Mn-oxidizing MS-1 4°C cultures began to form aggregates once they reached stationary phase (data not shown), so much of this decrease in optical density may be due to this aggregation.

**TABLE 5:** Growth rates and doubling times

	4°C		14°C		24°C	
	Growth rate (h^−1^)	Doubling time (h)	Growth rate	Doubling time	Growth rate	Doubling time
*P. putida* GB-1	0.04 ± 0.004	24.54 ± 2.14	0.51 ± 0.03	1.97 ± 0.12	1.31 ± 0.05	0.76 ± 0.03
*Pseudomonas* sp. DSV-1	0.12 ± 0.001	8.10 ± 0.09	0.44 ± 0.001	2.28 ± 0.004	0.95 ± 0.01	1.05 ± 0.01
*Pseudomonas* sp. MS-1	0.13 ± 0.01	7.51 ± 0.56	0.46 ± 0.01	2.19 ± 0.05	0.84 ± 0.06	1.20 ± 0.09

### *Pseudomonas* MS-1 and DSV-1 oxidize Mn at low temperature

**Fig 5:**
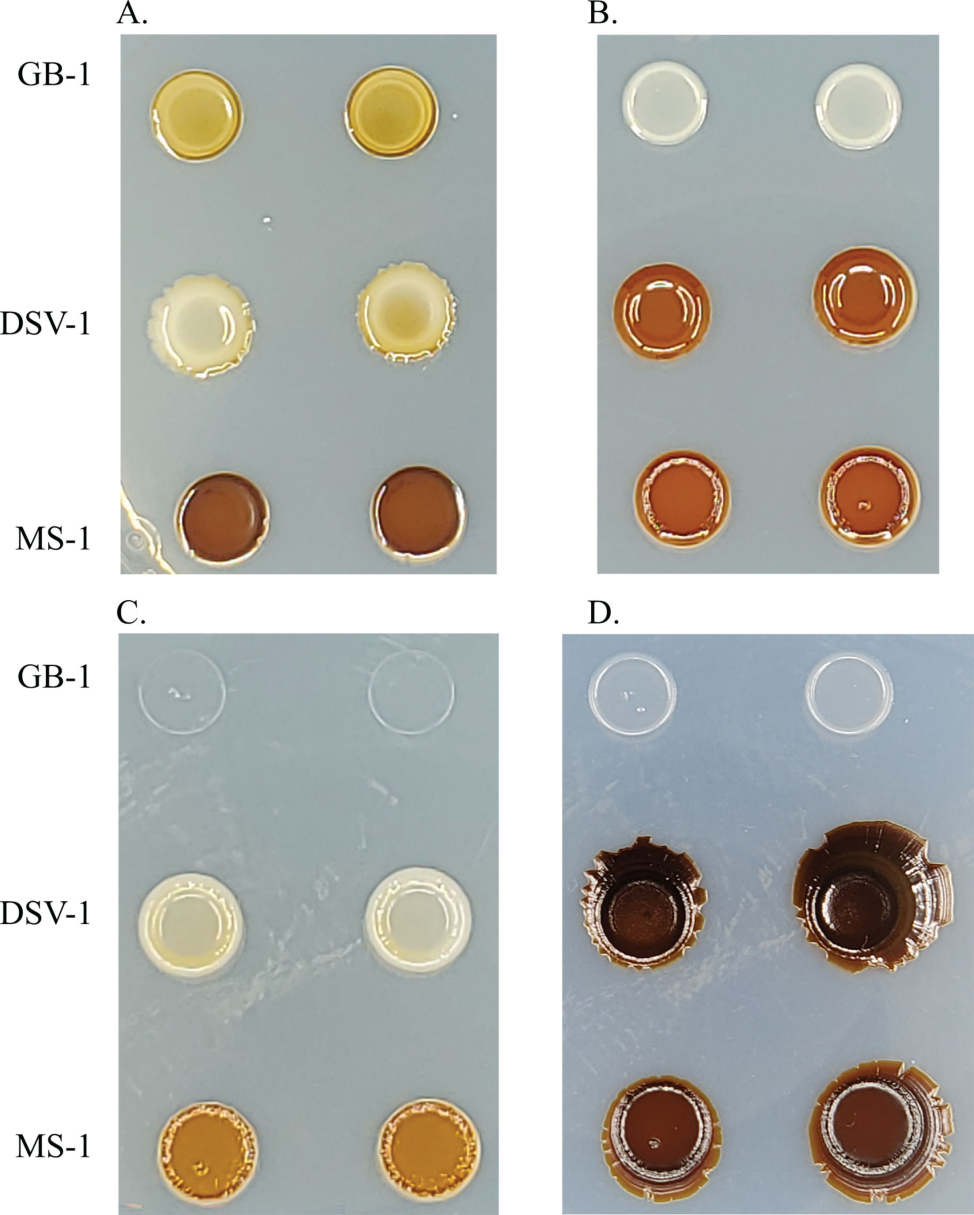
Effect of temperature on Mn(II) oxidation. Strains were incubated on Lept for 5 days at (A) 24°C, (B) 14°C, and (C) 4°C. (D) Plate C after 10 months at 4°C.

The growth curve experiments were performed in the presence of reduced Mn, so it was possible to observe that all three strains accumulated Mn oxides during the course of the experiment (data not shown). To verify this observation, each strain was incubated on solid Lept media at 24°C, 14°C, and 4°C (Fig. 5). After 5 days, at 24°C, all three strains grew and oxidized Mn, as seen by the brown colony color. At 14°C, again all three strains oxidized Mn, with GB-1 producing a lighter brown color than MS-1 or DSV-1. At 4°C, both MS-1 and DSV-1 produced brown colonies, but GB-1 produced barely detectable growth. After 10 months, GB-1 had still failed to form detectable Mn oxides, while DSV-1 and MS-1 continued to grow and accumulate Mn oxides.

### Genetic manipulation of DSV-1 and MS-1

The ability to genetically manipulate DSV-1 and MS-1 would make it possible to use these strains to investigate low-temperature Mn oxidation and generate strains optimized for bioremediation at low temperature. As a first step, we screened both strains for antibiotic sensitivity. DSV-1 and MS-1 are both resistant to ampicillin and penicillin but sensitive to gentamicin and kanamycin (data not shown). The selective medium Pseudomonas Isolation Agar (PIA, CRITERION, Hardy Diagnostics) is often used to isolate the *Pseudomonas* strains from environmental samples and during triparental mating (23). The basis of this selection is the presence of the broad-spectrum antimicrobial drug triclosan, which inhibits fatty acid synthesis. *Pseudomonas* spp. are naturally resistant to triclosan due to the presence of the FabV alternative fatty acid synthesis enzyme (33). However, neither DSV-1 nor MS-1 possesses a *fabV* homolog in their genomes, and neither strain can grow on PIA (data not shown).

Conjugation with *E. coli* is routinely used to introduce foreign DNA into *P. putida* GB-1 (22, 23). To demonstrate that conjugation can be used to move plasmids into DSV-1 and MS-1, we performed triparental conjugation to move the plasmids pBR322MCS-5 and pUCP22 (Table 7) into both strains. These plasmids both carry *aacC1*, the gentamicin-resistant marker gene; the successful transfer of the plasmid into DSV-1 and MS-1 was detected by the ability of the conjugants to grow on media containing gentamicin (data not shown). *P. putida* GB-1 can be made chemically competent and made to take up plasmids by heat shock transformation (23); a similar approach was successful with MS-1 but has not yet been tried with DSV-1.

To demonstrate the ability to generate mutations in the DSV-1 and MS-1 genomes, we conjugated into each strain the plasmid pRL27, which encodes the Tn5 transposon carrying a Kan^R^-resistant gene marker (Table 7). This plasmid has an *ori*R6K origin of replication and therefore requires the presence of the *pir* gene on the chromosome in order to be maintained as a plasmid (34). Since DSV-1 and MS-1 lack the *pir* gene, the only way to obtain Kan^R^ colonies after conjugation is if the Tn5 transposon carrying Kan^R^ has transposed into the chromosome. The ability to isolate Kan^R^ colonies after conjugation into DSV-1 and MS-1 (data not shown) therefore demonstrates that these strains can be manipulated by insertion of Tn5 into the chromosome.

**Fig 6:**
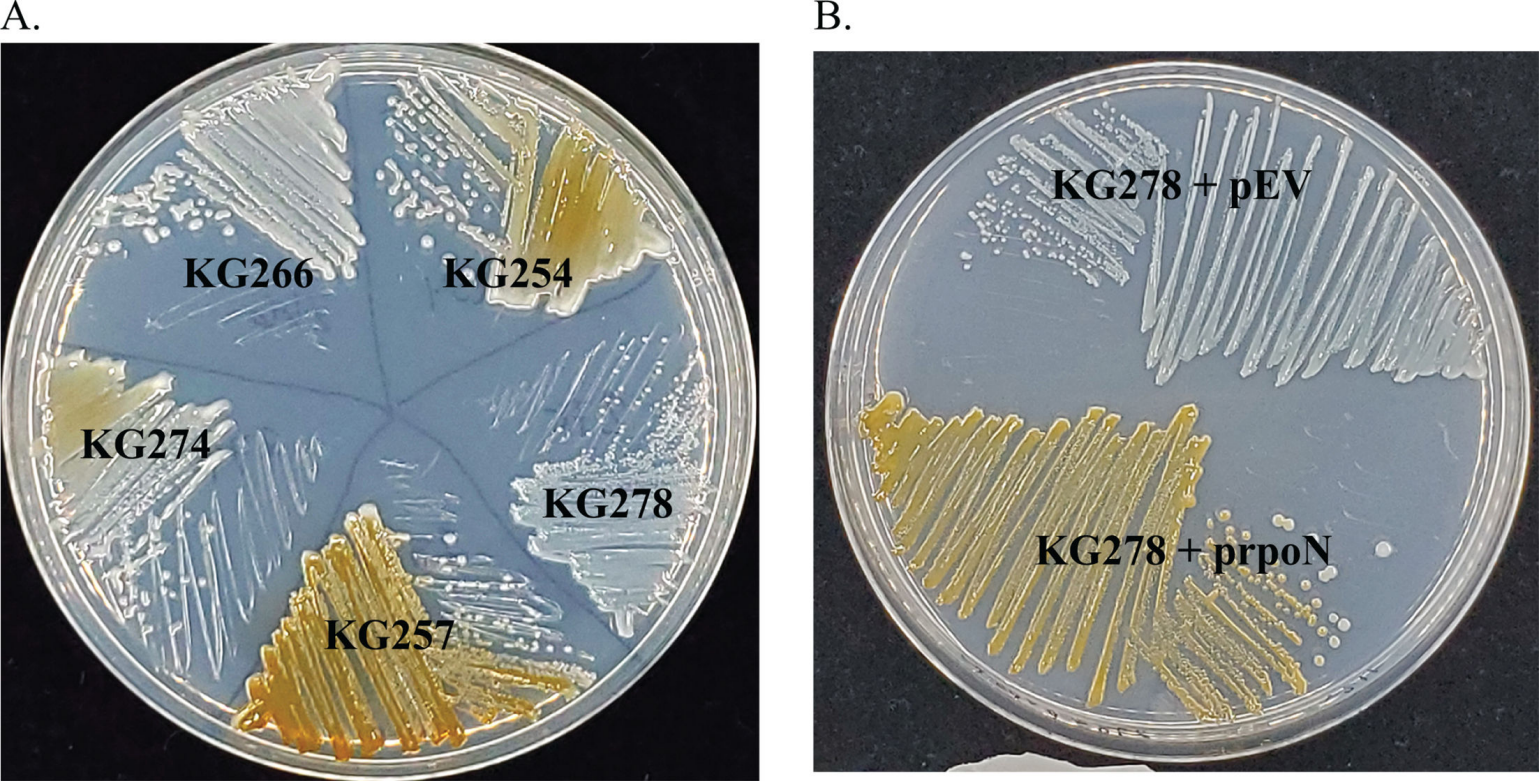
Generation of strains with altered Mn(II) oxidation. (A) Wild-type and Tn5 mutant strains, 2 days at 24°C on Lept. (B) KG278 with empty vector (EV) and with plasmid-carrying *rpoN* (prpoN), 2 days at RT on Lept.

After successfully isolating kanamycin-resistant colonies, the colonies were screened for their Mn oxidation phenotype, and 13 mutant isolates were identified with altered Mn oxidation activity (11 in the DSV-1 strain and 2 in MS-1). Mapping the site of insertion revealed that several different genes had been targeted by transposition of Tn5 (Table 6). The oxidation phenotypes ranged from slight increase (KG271) to slight decrease (KG274) to no oxidation (KG272, 277, and 278; Fig. 6A). In the non-oxidizing strain KG278 (Fig. 6A), the transposon was inserted into the gene *rpoN* (Table 8), which encodes the alternative sigma factor σ^54^. To verify that the oxidation defect of KG278 is due to the *rpoN*::Tn5 mutation, complementation was performed using a plasmid carrying the *P. putida* GB-1 *rpoN* gene (pKG228, Table 7). Complementation was successful; pKG228 restored Mn oxidation to *rpoN*::Tn5 (Fig. 6B).

**TABLE 6:** Tn5 mutations in DSV-1 and MS-1

Strain name	Parent strain	Gene disrupted (gene ID#)	Predicted function	Mn oxidation phenotype
KG266	DSV-1	Thiol-disulfide isomerase/thioredoxin; 2986951652	CCM^*a*^	Decreased
KG267	DSV-1	Thiol-disulfide isomerase/thioredoxin; 2986951652	CCM	Decreased
KG268	DSV-1	Thiol-disulfide isomerase/thioredoxin; 2986951652	CCM	Decreased
KG269	DSV-1	Thiol-disulfide isomerase/thioredoxin; 2986951652	CCM	Decreased
KG270	DSV-1	N-acetyl-gamma-glutamyl-phosphate reductase, 2986950346	Arginine biosynthesis	Slight decrease
KG275	DSV-1	Acetylglutamate kinase, 2986950012	Arginine biosynthesis	Slight decrease
KG276	DSV-1	N-acetyl-gamma-glutamyl-phosphate reductase, 2986950346	Arginine biosynthesis	Slight decrease
KG271	DSV-1	23S rRNA gene; 2986950353	Translation	Increased
KG272	DSV-1	tRNA uridine 5-carboxymethylaminomethyl modification enzyme, 2986954883	Translation	No oxidation
KG273	DSV-1	23S rRNA gene; 2986950353	Translation	Increased
KG274	DSV-1	Fumarate hydratase class I; 2986953570	TCA cycle	Slight decrease
KG277	MS-1	Multi-component K^+^:H^+^ antiporter subunit A; 2986958496	Transport	No oxidation
KG278	MS-1	RNA polymerase sigma-54 factor; 2986956421	Gene expression, sigma54 regulon	No oxidation

^*a*^ Cytochrome c maturation.

**TABLE 7:** Plasmids and strains

Strain or plasmid	Genotype, characteristics, or construction	Antibiotic resistance^*a*^	Source or reference
*E. coli* strains			
GT155	*F*¯ *mcr*A ∆(*mrr-hsdRMS-mcr*BC) F80*lacZ*∆M15 ∆l*acX*74 *rec*A1 *end*A1 ∆*dcm, uidA(∆MluI)::pir-116, ∆sbcC-sbcD*	N/A	InvivoGen
DH5α		N/A	
*Pseudomonas* strains			
*P. putida* GB-1	Manganese oxidizer, wild type	Amp^R^	(35)
*Pseudomonas* sp. MS-1		Amp^R^	This work
*Pseudomonas* sp. DSV-1		Amp^R^	This work
KG266	*Pseudomonas* sp. DSV-1, Ga0589516_01_1717614_1718486::Tn5	Amp^R^, Kan^R^	This work
KG274	*Pseudomonas* sp. DSV-1, Ga0589516_01_3718433_3719956::Tn5	Amp^R^, Kan^R^	This work
KG278	*Pseudomonas* sp. MS-1, Ga0589517_01_982383_983852::Tn5	Amp^R^, Kan^R^	This work
Plasmids			
pRL27	Tn5	Kan^R^	(34)
pRK2013	ColE1 replicon, *mob* RK2, *tra* RK2	Kan^R^	(36)
pBBRMCS1-5	Broad host range expression vector	Gm^R^	(37)
pUCP22	Broad host range expression vector	Amp^R^, Gm^R^	(38)
pKG228	*P. putida* GB-1 *rpoN* gene cloned into pUCP22	Amp^R^, Gm^R^	This work

^*a*^ Amp^R^, ampicillin resistance; Kan^R^, kanamycin resistance; Gm^R^, gentamicin resistance.

## DISCUSSION

In this work, novel MnOB were isolated from compost, supporting a possible role for Mn oxidation in the breakdown of complex organic molecules. Both MS-1 and DSV-1 were shown to grow and oxidize Mn at low temperature. A complete genome sequence and phylogenetic characterization showed these strains to be closely related to *Pseudomonas psychrophila* but genetically distinct. Therefore, they have been named *Pseudomonas* sp. DSV-1 and MS-1. Both strains are amenable to genetic manipulation and carry, in their genomes, genes homologous to those previously identified as important for Mn oxidation in *P. putida* GB-1.

### Low-temperature growth

DSV-1 and MS-1 both grow well at 4°C but grow faster at 24°C (Fig. 4; Table 7). Psychrophilic organisms are commonly defined as those that grow best at temperatures below 20°C and thus are confined to environments that are continuously cold. Conversely, psychrotrophic or psychrotolerant organisms grow best at 20°C or above but grow well at temperatures below 20°C (39). Given this definition, DSV-1 and MS-1 are best described as psychrotolerant. Cold-tolerant species have previously been identified in the genus *Pseudomonas* from environments as diverse as Antarctic sea ice and food spoilage (40, 41). For example, *Pseudomonas psychrophila* HA-4 was isolated by its ability to degrade the antibiotic sulfamethoxazole at low temperature (42), and *Pseudomonas fragi* strains were isolated from the leaves of cold-adapted plants (43). MS-1 and DSV-1 are closely related to *P. fragi* and *P. psychrophila* (Fig. 2). Thus, the cold tolerance of *Pseudomonas* strains isolated from a compost pile located outside in Minnesota in winter was not unexpected.

### Low-temperature Mn oxidation

While MnOB have generally been characterized as mesophiles (35, 44), Mn oxidation at low temperature has been observed before. *Brevibacillus brevi* MO1 has been shown to oxidize Mn at 4°C but not to the same extent as it does at 37°C (45). *Arthrobacter* sp. NI-2 normally oxidizes Mn at 30°C; a mutation in this strain allows it to oxidize at 10°C (46). The dormant spores of *Bacillus* sp. SG-1 are capable of producing Mn oxides over a very wide range of temperatures, from 0°C to 80°C (47). *Pseudomonas* sp. MOB-449 grows well and exhibits its maximum Mn oxidation capacity at 18°C (48). At this low temperature, Mn stimulates biofilm growth and expression of the c-type cytochrome biosynthesis enzyme CcmE, leading to the proposal that the Mn oxidation supplements the cell’s energy needs (49). Thus, while *P. psychrophila* DSV-1 and MS-1 are not the only MnOB capable of low-temperature Mn oxidation identified so far, they are the first characterized that actively grow and robustly oxidize at temperatures as low as 4°C.

### Conservation of Mn oxidation mechanism

Many of the genes identified as playing a role in Mn oxidation in *P. putida* GB-1 are also present in DSV-1 and MS-1. Each has orthologs to the Mn oxidase genes *mnxG* and *mcoA* but lack clear orthologs to *mopA* (Table 4). This suggests that Mn oxidation in these strains depends on the multi-copper oxidases MnxG and McoA but not the heme peroxidase MopA. DSV-1 and MS-1 also carry orthologs to the Mnx two-component regulatory pathway comprising MnxS1, MnxS2, and MnxR. MnxR in *P. putida* GB-1 is required for Mn oxidation and is predicted to be a σ^54^-dependent transcription factor, based on its domain composition (23). The MnxR orthologs in MS-1 and DSV-1 are also predicted to contain σ^54^ interaction domains. This suggests that the expression of Mn oxidation genes is driven by RNA polymerase containing σ^54^ in all three strains. Supporting this conclusion, a Tn5 insertion in the predicted *rpoN* gene of MS-1 resulted in a strain completely defective for Mn oxidation when assayed on solid media (Fig. 6) and in liquid culture (data not shown). This oxidation defect could be complemented with the GB-1 *rpoN* gene, reinforcing the conclusion that Mn oxidation in this strain is σ^54^ dependent.

Previous work has shown that Mn oxidation in *P. putida* GB-1 can be disrupted by Tn5 insertions in genes encoding components of the TCA cycle, including the succinate dehydrogenase complex (*sdhABC*), lipoate acetyltransferase (*aceA*), and isocitrate dehydrogenase (*icd*) (50). Insertion of Tn5 into the fumarate hydratase class I gene of DSV-1 resulted in moderately decreased Mn oxidation (KG274, Fig. 6); fumarate hydratase catalyzes the conversion of fumarate to malate in the TCA cycle. KG266–269 all have Tn5 inserted in a predicted thiol-disulfide isomerase (Table 8). In *Bradyrhizobium japonicum*, a similar protein called TlpA is involved in cytochrome c oxidase maturation (51). In DSV-1, the gene is in a putative operon between *dsbD* and *dsbG* genes, raising the possibility of polar effects on these neighboring genes. In *Shewanella oneidensis*, DsbD facilitates the transfer of electrons to the protein CcmG during the cytochrome c maturation (CCM) process (52). In *P. putida* MnB1, the CCM genes *ccmA, E,* and *F* have previously been identified as playing a role in Mn oxidation (50), and CcmE has been implicated in low-temperature Mn oxidation in *Pseudomonas* sp. MOB-449 (49). Thus, the function and regulation of Mn oxidation in the new isolates are likely similar to that in other Mn-oxidizing pseudomonads.

**TABLE 8:** Primer list

Name	Sequence	Reference
8F	5′-AGAGTTTGATCCTGGCTCAG-3′	(53)
519R	5′-GWATTACCGCGGCKGCTG-3′	(54)
tpnRL17-1	AACAAGCCAGGGATGTAACG	(34)
tpnRL13-2	CAGCAACACCTTCTTCACGA	(34)
ARB1	GGCCACGCGTCGACTAGTACNNNNNNNNNNTACNG	(55)
ARB2	GGCCACGCGTCGACTAGTACNNNNNNNNNNGATAT	(55)
ARB3	GGCCACGCGTCGACTAGTACNNNNNNNNNNACGCC	(55)
ARB4	GGCCACGCGTCGACTAGTAC	(55)
Tnp5IR-2R	GGTTGAGATGTGTATAAGAGACAG	(56)
rpoN_1-F	CGCGAATTCGCGAACAAGGTATTAAGCCC, incorporates an EcoRI recognition site upstream of *rpoN*	This work
rpoN_2-R	CGCTCTAGACGTGCATAAAGAAGCAGGTC, incorporates an XbaI recognition site downstream of *rpoN*	This work

### Low-temperature bioremediation

There are many potential applications for MnOB and biogenic Mn oxides in bioremediation. Cold-tolerant bacteria and their enzymes are also valuable tools for bioremediation and other industrial applications (42, 57, 58). Therefore, *Pseudomonas* ssp. MS-1 and DSV-1 expand the conditions under which MnOB can be used for bioremediation due to their ability to form Mn oxides at low temperature. Our preliminary results suggest the two strains differ in the effect of temperature on their ability to accumulate oxidized Mn. As judged by the intensity of brown oxides formed, MS-1 robustly formed Mn oxides at all three temperatures tested, while DSV-1 best formed oxides at the intermediate temperature of 14°C (Fig. 5). MS-1 also tolerates growth at temperatures above 24°C better than DSV-1 (data not shown), which suggests this strain will be the better target for bioremediation applications.

At cold temperatures, bacteria experience stress due to decreased membrane fluidity, decreased enzyme activity, altered redox state, and increased stability of RNA and DNA structures, which interfere with replication and gene expression (59–61). The MS-1 and DSV-1 genomes are very similar to one another (Tables 2 and 3); comparing these genomes may make it possible to determine the genetic basis for their differences in oxidation and temperature sensitivity phenotypes. Preliminary characterization of cold shock genes in GB-1, DSV-1, and MS-1 (Table 2 and data not shown) failed to reveal a genetic basis for the cold tolerance of DSV-1 and MS-1 since all three genomes possess six putative cold shock protein genes (*cspA*). Both strains can be made to take up foreign DNA by conjugation and transformation; they can express foreign genes from plasmids and can have their genomes mutated with a transposon. The apparent conservation of Mn oxidation and its regulation between the new isolates and the well-characterized MnOB *P. putida* GB-1 will guide future efforts to generate cold-tolerant strains optimized for Mn oxidation under various conditions.

## MATERIALS AND METHODS

### Media and culture conditions

Strains and plasmids used in this study are listed in Table 7. *Pseudomonas* strains were grown in LB or Lept liquid and solid media made according to the procedure of reference (25). Strains were grown at 24°C, 14°C, or 4°C. *Escherichia coli* strains were grown in LB medium at 37°C. The following concentrations of antibiotics were used: ampicillin (100 µg/mL), gentamicin (50 µg/mL), and kanamycin (30 µg/mL). For oxidation assays, MnCl_2_ was added to Lept medium at a final concentration of 100 µM. Phosphate-buffered saline was made according to standard protocols (62).

### Sample collection

Samples for cultivation were collected from a compost pile on the University of Minnesota, Morris campus that is composed of a 3:1 ratio of plant material to food waste (Ostby, Personal Communication). Samples were taken in February 2019 using sterile, plastic 50 mL tubes. The tubes were opened and immediately used to scoop material from the compost surface, ~15 cm, or ~30 cm below the surface. After collection, the tubes were sealed, immediately transported back to the lab, and stored at 4°C. Furthermore, 1 g of sample was incubated in PBS pH 7.3 for 5 min at RT, with shaking. The PBS/compost mixture was allowed to settle 10–15 min, and then 100 µL of the supernatant was spread onto Lept plates. After incubation at RT for 7 days, thousands of colonies were visible, with a subset of brown, putative Mn-oxidizing colonies. Mn oxidation was confirmed using a leucoberbelin blue spot test (25). LBB-positive colonies were selected and subcultured onto fresh Lept lates. After several rounds of re-streaking, DSV-1 and MS-1 were shown to be pure via microscopic observation.

### Identification of isolates by 16S rRNA sequencing

To obtain 16S amplicons from our bacterial sample, colony PCR was run using iProof High-Fidelity DNA Polymerase using the following concentrations of reagents: 200 µM dNTP mix, 1 µM forward primer, 1 µM reverse primer, 0.5 U of iProof High-Fidelity DNA Polymerase per 50 µL reaction, 10 µL of 5× iProof HF Buffer per 50 µL reaction, and 1 µL of overnight culture in NB broth as the DNA template source. Primers 8F and 519R (Table 8) were used to generate an ~500 bp amplicon. Reaction conditions were initial denaturation at 98°C for 3 min followed by 25 cycles of 98°C for 30 s, 55°C for 1 min, 72°C for 1 min, followed by 72°C for 5 min.

PCR amplicons were purified using DNA Clean & Concentrator-5 according to manufacturer’s instructions (Zymo Research, Irvine CA). The concentration of DNA samples was determined using a Qubit 3.0 Fluorometer from manufacturer Invitrogen (Carlsbad, CA). Furthermore, 50 ng of 500 bp length sequences and 200 ng of 1,500 bp sequences were added to new tubes for sequencing by the University of Minnesota Genomics Center (http://genomics.umn.edu/). Also included in the samples were 6.4 pmol of the appropriate primers (Table 2). Short amplicons were sequenced using 8F and 519R, while the long amplicons were sequenced with 8F, 519R, 1492R, 533F, and CDR (Table 2).

Amplicons of the 16S SSU gene were conjoined using GeneStudio (https://sourceforge.net/projects/genestudio/) to produce a consensus sequence of 1,467 bp. This consensus sequence was then used to query the 16S ribosomal RNA (bacteria and archaea) database using BLASTN (63, 64).

### DNA extraction and genome sequencing

Cultures were grown on solid R2A medium, and a single colony was transferred to 10 mL of tryptic soy broth and grown for 48 h with shaking. Five milliliters of each culture were then centrifuged for 10 min at 2,000 × *g* in a swinging bucket rotor, and the supernatant was removed. The cell pellets were then resuspended in 0.5 mL of sterile PBS pH 7.4 (Gibco-Thermo Fisher, Waltham MA), and DNA was extracted using the QIAamp UCP Pathogen Kit (QIAGEN, Germantown MD) following the standard protocol with the final elution in molecular biology grade water. Purified DNA was quantified using a Qubit 4 fluorometer using dsDNA HS Assay (Invitrogen-Thermo Fisher, Waltham MA). Illumina sequencing was performed using the Nextera DNA Flex Library prep following the standard protocol and sequenced with a 600-cycle MiSeq v3 Reagent Kit (Illumina, San Diego, CA). Long-read sequencing was performed using a 1D2 R9.2 Sequencing Kit on an Oxford Nanopore Minion sequencer (Oxford Nanopore, New York, NY). Read coverage was approximately 120× for Illumina sequencing and 30× for Nanopore sequencing. Illumina reads were trimmed for quality, and adapters were removed using Trimmomatic V0.39 (65). Illumina and Nanopore reads were then used to assemble the genomes using the Unicycler assembly pipeline V0.4.8 (66) with Spades V3.13.0 (67).

### Generation of *Pseudomonas* phylogenetic tree

Assembled genomes for 88 *Pseudomonas* species were downloaded from NCBI, and 4 housekeeping genes (16S rRNA, *rpoB*, *rpoD*, and *gyrB*) were extracted from each assembled genome [as in reference (28)]. These four genes were concatenated and aligned with *Cellvibrio japonicus* as an outgroup using default parameters in MAFFT [version 7; (68)]. This alignment was used to build a phylogenetic tree with RAxML [v. 8.2.11; (69)]. The rapid bootstrapping and search for best scoring ML tree approach was used with 1,000 replicates, the input was partitioned by each gene, and a GTR Gamma nucleotide model was implemented. All of the above took place within the Geneious Prime (v. 2019.1.1) interface.

### Growth curves, growth rate, and doubling times

Growth rates and doubling times were calculated using spectrophotometry at a wavelength of 600 nm. Cultures of GB-1, DSV-1, and MS-1 were grown overnight in 5 mL of Lept media with continuous agitation at 240 rpm at a temperature of 24°C. Subcultures of each strain were prepared in triplicate by diluting the overnight cultures 100-fold into 50 mL of Lept media. These subcultures were then grown at 24°C, 14°C, and 4°C with continuous agitation at 240 rpm. Furthermore, 1 mL samples were taken periodically to determine optical density using a spectrophotometer.

### Transposon mutagenesis

The plasmid carrying the transposon Tn5, pRL27, was moved into DSV-1 and MS-1 by triparental conjugation (23) and transconjugants selected by plating on LB containing 30 µg/mL kanamycin and 100 µg/mL ampicillin. Colonies were replica plated onto solid Lept media to screen for variations in the manganese oxidation phenotype. Selected MS-1 and DSV-1 mutants were streaked for single colonies on Lept media and compared to wild type to confirm the variation in their manganese-oxidizing capabilities.

### Mapping site of transposon insertion

Some Tn5 insertion sites were mapped according to the protocol of reference (24) with the following exceptions. Genomic DNA was isolated using the Wizard Genomic DNA Purification Kit (Promega, Madison, WI). Five micrograms of purified gDNA were digested with BamHI in a 50 µL reaction overnight at 37°C. The digested DNA was ethanol precipitated, and 100 ng was ligated using T4 DNA ligase (New England BioLabs, Ipswich, MA) in a 20 µL reaction overnight at room temperature. The ligation reactions were then transformed into *E. coli* GT115 commercially made competent cells (Invivogen CHEMICOMP GT115, Fisher Scientific). LB agar with Km was used to select *E. coli* cells transformed with the plasmid containing Tn5 and the BamHI fragment of the genome. Plasmids were purified from Km^R^ colonies using the QIAprep Spin Miniprep Kit (Qiagen,Valencia, CA). Purified plasmids were sent to be sequenced at Functional Biosciences (https://functionalbio.com/) using primers tpnRL17-1 and tpnRL13-2 (Table 2). The sequenced genes were identified using a BLAST search against the relevant genome database on the Integrated Microbial Genomes website (http://img.jgi.doe.gov/) (70).

The remaining Tn5 insertion sites were mapped using an arbitrary PCR approach (55). Genomic DNA was prepared as above. Three reactions were performed for each mutant, each using tpnRL17-1 as the forward primer, and ARB1, ARB2, or ARB3 as the reverse primer (Table 2). Furthermore, 1 µL of genomic DNA was used as template, 1× Promega GoTaqG2 Hot Start Green Master Mix, and 0.8 µM final concentration primers in total volume of 25 µL. PCR conditions were as follows: 1 cycle of 95°C 5 min, 6 cycles of 94°C 30 s, 30°C 30 s, 72°C 2 min followed by 30 cycles of 94°C 30 s, 45°C 30 s, 72°C 2 min followed by 72°C 5 min and then stored at 4°C. One microliter of this reaction was used as template in a second PCR with ARB4 and tnp5IR-2R as the primers, 0.8 µM final concentration, and 1× Promega GoTaq G2 Hot Start Green Master Mix in a total volume of 30 µL. PCR conditions were as follows: 1 cycle of 95°C 5 min, 30 cycles of 94°C 30 s, 55°C 30 s, 72°C 2 min followed by 72°C 5 min and then stored at 4°C. Reactions were separated on a 1% low melt agarose gel; the prominent band from each reaction was excised using a razor blade and stored at 4°C. One microliter of liquid from the excised band was used as template for a third round of PCR with the same primers and conditions as the second PCR. The DNA from both the excised gel band and the third PCR was cleaned using the GeneJET Gel Extraction and DNA Cleanup Micro Kit (ThermoScientific) and was sent to Functional Biosciences (https://functionalbio.com/) to be sequenced using primer Tnp5IR-2R (Table 2).

### Construction of the rpoN plasmid

The *rpoN* gene was PCR amplified from *Pseudomonas putida* GB-1 using primers rpoN_1 F and rpoN_2 R (Table 2), with a high-fidelity DNA polymerase (Phusion HotStart high-fidelity DNA polymerase). The resulting PCR product was cloned into pJET1.2/blunt (CloneJet PCR Cloning Kit; Fermentas, Glen Burnie, MD). The genes were subsequently subcloned into the broad host-range plasmid pUCP22 (Table 1) using the EcoRI and XbaI restriction enzyme recognition sites engineered into the amplification primers, and the presence of the insert in the resulting plasmid was confirmed by restriction digest. The genes inserted into pUCP22 are expressed constitutively from the plasmid-borne promoter P_lac_.

### Transformation of *Pseudomonas* sp. MS-1 and derivatives

*Pseudomonas* sp. MS-1 and the MS-1 *rpoN*::Tn5 mutant were made competent as follows. Bacteria were grown overnight in LB and subsequently diluted 25-fold into fresh LB and grown at room temperature for 4 h. Furthermore, 2 mL of cells was pelleted by centrifugation at 12,000 × *g* for 1 min and then washed with 1 mL of ice cold 0.1 M CaCl_2_. Cells were then pelleted and resuspended in 1 mL ice cold 0.1M CaCl_2_ and incubated on ice for 30 min. Finally, cells were pelleted and resuspended in 100 µL ice cold CaCl_2_. Transformation was performed by adding 2 µL plasmid to the cells and incubating on ice for 30 min. Next cells were exposed to heat shock for 90 s at 37°C and then returned to ice for 2 min. Then, 400 µL SOC medium was added to each transformation, which were then incubated at room temperature with shaking for 1 h. Finally, the entire transformation was plated onto LB_Gm_ plates and incubated at room temperature.
